# Radiation and Drought Impact Residual Leaf Conductance in Two Oak Species With Implications for Water Use Models

**DOI:** 10.3389/fpls.2020.603581

**Published:** 2020-11-27

**Authors:** Haiyan Qin, Carles Arteaga, Faqrul Islam Chowdhury, Elena Granda, Yinan Yao, Ying Han, Víctor Resco de Dios

**Affiliations:** ^1^School of Life Sciences and Engineering, Southwest University of Science and Technology, Mianyang, China; ^2^Department of Crop and Forest Sciences, University of Lleida, Lleida, Spain; ^3^Institute of Forestry and Environmental Sciences, University of Chittagong, Chattogram, Bangladesh; ^4^Department of Life Sciences, University of Alcalá, Alcalá de Henares, Spain; ^5^Joint Research Unit CTFC-AGROTECNIO, Universitat de Lleida, Lleida, Spain

**Keywords:** cuticular conductance, stomatal conductance, night conductance, dark respiration, drought, shade

## Abstract

Stomatal closure is one of the earliest responses to water stress but residual water losses may continue through the cuticle and incomplete stomatal closure. Residual conductance (*g*_*res*_) plays a large role in determining time to mortality but we currently do not understand how do drought and shade interact to alter *g*_*res*_ because the underlying drivers are largely unknown. Furthermore, *g*_*res*_ may play an important role in models of water use, but the exact form in which *g*_*res*_ should be incorporated into modeling schemes is currently being discussed. Here we report the results of a study where two different oak species were experimentally subjected to highly contrasting levels of drought (resulting in 0, 50 and 80% losses of hydraulic conductivity) and radiation (photosynthetic photon flux density at 1,500 μmol m^–2^ s^–1^ or 35–45 μmol m^–2^ s^–1^). We observed that the effects of radiation and drought were interactive and species-specific and *g*_*res*_ correlated positively with concentrations of leaf non-structural carbohydrates and negatively with leaf nitrogen. We observed that different forms of measuring *g*_*res*_, based on either nocturnal conductance under high atmospheric water demand or on the water mass loss of detached leaves, exerted only a small influence on a model of stomatal conductance and also on a coupled leaf gas exchange model. Our results indicate that, while understanding the drivers of *g*_*res*_ and the effects of different stressors may be important to better understand mortality, small differences in *g*_*res*_ across treatments and measurements exert only a minor impact on stomatal models in two closely related species.

## Introduction

Plant transpiration through stomatal pores and leaf cuticles dominates global evapotranspiration (Hetherington and Woodward, 2003). As water stress intensifies under global warming, there is an increasing interest toward understanding ecological variation in residual leaf conductance (*g*_*res*_). After stomatal closure, water loss continues until mortality due to a mixture of cuticular water loss and incomplete stomatal closure (residual conductance; Blackman et al., 2016; Martin-Stpaul et al., 2017).

Studies addressing ecological and physiological variation in the drivers of residual conductance are currently rare (Heredia-Guerrero et al., 2018). According to a recent review on this topic (Duursma et al., 2019), only 10 studies have addressed the effect of drought on *g*_*res*_ and, from those, only 4 had been performed on trees. Consequently, multi-factorial studies addressing ecological variation in residual conductance are much needed to understand its variation. For instance, while shade and drought are both known to decrease residual conductance (Boyer et al., 1997; Shepherd and Wynne Griffiths, 2006), it is currently unknown whether the effect of both stressors would be additive or interactive. However, the effects of residual conductance on mortality have been documented to be dramatic: time to mortality nearly doubles if *g*_*res*_ declines from 4 to 2 mmol m^–2^ s^–1^ (Duursma et al., 2019).

Understanding the physiological and ecological drivers of *g*_*res*_ has been the topic of some discussion (Riederer and Müller, 2007; Fernández et al., 2017). Some studies report that variations in the degree of sclerophylly (as indicated by leaf mass area) would increase *g*_*res*_ because leaves that are more scleromorphic will show thicker cuticles, but other work has demonstrated that changes in wax composition may compensate for such effect (Bueno et al., 2020). Another alternative, explored to a lesser degree, is that further reductions in *g*_*res*_ may be inhibited by changing carbohydrate allocation priorities (Zhang et al., 2020). In other words, as non-structural carbohydrate reserve pools deplete, cuticle production to prevent cuticular water losses may be limited by NSC availability.

Understanding variation in residual conductance is also necessary for models of water use (Leuning, 1995; Barnard and Bauerle, 2013; De Kauwe et al., 2015), where residual conductance acts as the intercept of commonly used stomatal models (*g*_*int*_). The most common stomatal models being used in land surface models are Ball-Berry model types, which have the general form:

(1) $$g_{\text{s}}=g_{\text{int}}+mA/C_{\text{a}}\text{f}\left(\text{D}\right)$$

Where *g*_*s*_ is stomatal conductance, *A*, *C*_*a*_, and *D* represent photosynthesis, ambient CO_2_ concentration and vapor pressure deficit, respectively, and *m* is the slope parameter. When *g*_*int*_ is estimated through regression fitting, it may either be equal to 0, which creates problems because then the ratio of intercellular to ambient CO_2_ (*C*_*i*_/*C*_*a*_) does not vary with light (Collatz et al., 1991; Leuning, 1995; Duursma et al., 2019), or it may be negative, which is nonsensical.

There are at least two possible definitions of *g*_*int*_: (1) *g*_0_, which represents the lowest conductance reached as photosynthesis tends to 0 because light declines (Leuning, 1995; Barnard and Bauerle, 2013); (2) *g*_*min*_, which refers to the residual conductance after (complete or not) stomatal closure under strong water stress (Duursma et al., 2019). We note that some studies use *g*_*min*_ and *g*_*res*_ interchangeably but, for clarity, we will differentiate them here as previously defined.

The problem then becomes how to measure *g*_0_ and *g*_*min*_. *g*_0_ could simply be measured as daytime conductance (*g*_*d*_) under low light in non-droughted plants and, similarly, *g*_*min*_ could similarly be measured from *g*_*d*_ in droughted plants (for as long as photosynthesis tends to zero, in both cases; Barnard and Bauerle, 2013; Duarte et al., 2016). Additionally, residual conductance has most often been measured by monitoring the water mass loss in detached leaves (*g*_*MLD*_; Kerstiens, 1996; Schuster et al., 2017). *g*_0_ and *g*_*min*_ could thus be measured with this method by comparing *g*_*MLD*_ in plants that have grown under strong light limitation or under strong water limitation, respectively. The problem with this approach, however, is that some acclimation responses (particularly in response to low radiation) could alter leaf morphology and it is unclear whether *g*_0_ measured through *g*_*MLD*_ after low light acclimation would be representative of that in plants without acclimation to low radiation.

An alternative would be to use nocturnal conductance (*g*_*n*_; Lombardozzi et al., 2017) in non-droughted and droughted plants. An advantage would be that photosynthesis would always be zero in this case. Duursma et al. (2019), however, proposed that *g*_*n*_ should not be used given the evidence of active regulation of stomatal conductance overnight (Resco De Dios et al., 2019), and that the drivers of nocturnal conductance could differ from those driving daytime conductance (Ogle et al., 2012). Amongst other processes, *g*_*n*_ varies through time due to circadian regulation (Resco De Dios et al., 2015). However, *g*_*n*_ often retains some sensitivity to *D* such that maximum stomatal closure and, potentially, residual conductance, may be achieved at lower *D* than during the daytime (Barbour and Buckley, 2007). One could thus hypothesize that measurements of *g*_*n*_ under high *D* may be indicative of *g*_*res*_.

Regardless of how *g*_0_ and *g*_*min*_ are estimated, Duursma et al. (2019) proposed to replace Eq. 1 by:

(2) $$g_{\text{s}}=\text{max}\left[\text{max}\left(g_{0},g_{\text{min}}\right),mA/C_{\text{a}}\text{f}\left(\text{D}\right)\right]$$

That is, according to Eq. 2, residual conductance would not be added to the right-hand term of Eq. 1. Instead, one would use measured residual conductance (the maximum between *g*_0_ and *g*_*min*_) as an actual minimum (De Kauwe et al., 2015). However, this formulation has not yet been tested against data and, therefore, we do not yet know whether it enhances the predictive power of stomatal models.

Here we evaluate the effects of shade and water stress on *g*_*res*_ across two different oak species, the deciduous *Quercus faginea* and the sclerophyll *Q. ilex*. These two species are common in the calcareous soils from Spain and the Western Mediterranean Basin and we expected conductance to be significantly lower in *Q. ilex*, a species with a more conservative water use. More specifically, we sought to test: (1) how do drought and shade interact to affect *g*_*res*_? and (2) what are some of the possible mechanisms underlying variation in *g*_*res*_ across drought and shade treatments? Because *g*_*MLD*_ is probably the most accepted method to measure residual conductance, here we focused on *g*_*MLD*_. In particular, we addressed whether *g*_*MLD*_ would be driven by water stress (as indicated by water potential), NSC, LMA, or nitrogen concentration (*N*_*mass*_, an indicator of photosynthetic capacity), and whether *g*_*res*_ could limit respiration. We also sought to understand: (3) whether we obtain different values of *g*_*res*_ depending upon whether it is measured from *g*_*MLD*_, *g*_*n*_, and *g*_*d*_; and (4) how do we incorporate residual conductance into Ball-Berry type stomatal models and what are the consequences of variation in *g*_*res*_ across treatments and types of measurements for coupled leaf gas exchange models?

## Materials and Methods

### Experimental Design and Growing Conditions

The experiment was performed at the experimental fields from the University of Lleida (Spain; 41.62 N, 0.59 E). We built a rain-out shelter covered by clear polyethylene plastic, which is commonly used in greenhouse building. Half of the structure received solar radiation (sun treatment), with a maximum photosynthetically active radiation (PAR) of 1,500 μmol m^–2^ s^–1^. The other half was covered by a dense shading cloth (shade treatment) with a maximum PAR of 35–45 μmol m^–2^ s^–1^, which was near the light compensation point in this species (*data not shown*). The structure had openings on both sides to increase ventilation. Temperature inside the rain out shelter was 3°C higher than outside, but differences between the sun and shade treatment were negligible. Full details on the infrastructure have been provided elsewhere (Resco De Dios et al., 2020).

For this study we sourced 2 year-old seedlings from local nurseries (*n* = 120). The ecotypes for both species were original from the mountain range of the Iberian System. Plants were grown in 11 L cubic pots (20 cm × 20 cm × 27.5 cm). The substrate used was Humin Substrat Neuhaus N6 [Klasman-Deilmann GmbH, Geeste, Germany], a commercial potting mix. Pots were regularly fertilized with a slow release NPK MgO fertilizer (17-09-11-2, Osmocote Universal, KB, Ecully, France) and daily watered to field capacity until treatment implementation. The position of the pots was randomly shifted every other week.

The plants grew for 4 months into the rainout shelter before experiment inception in July 2017. That is, they developed new leaves under the assigned experimental light conditions. Although we cannot discard legacy effects from the previous growing season in the nursery (Aranda et al., 2001), all plants were treated equally.

We performed a full factorial experiment with the plants experiencing two light treatments crossed with three water stress treatments. Half of the plants grew under the sun treatment and the other half under the shade treatment, as previously described. We implemented three different water stress treatments using three different levels of percent loss of hydraulic conductivity (PLC): (i) P_0_, where plants were irrigated at field capacity; (ii) P_50_, where plants experienced 50% losses in hydraulic conductivity and which represents an important stress; and (iii) P_80_, where the plant experienced 80% losses in hydraulic conductivity, which represents a major stress and potentially mortality (Resco et al., 2009).

We kept plants at field capacity until treatment implementation. We then stopped watering and allowed plants to dehydrate and we measured midday stem water potential (Ψ_*md*_) every other day in a subset of plants (*n* = 5). The levels of PLC were controlled from the relation between midday shoot water potential (Ψ_*md*_) and PLC values reported previously in vulnerability curves from *Quercus faginea Lam.* (Esteso-Martínez et al., 2006) and *Quercus ilex L.* (Peguero-Pina et al., 2014). Shoot Ψ_*md*_ was regularly measured during treatment implementation with a pressure bomb (PMS 1000, PMS Instruments, Albany, Oregon) after clipping the sample and allowing for equilibration in the dark for ∼30 min. Once plants reached the target PLC, we kept soil moisture constant at that level for 2 weeks. This was achieved by weighing a subset of pots (*n* = 5 per each treatment) and adding back the water that had evaporated every day. We also measured native embolism to test the actual levels of PLC that we achieved in every treatment, as previously published (Resco De Dios et al., 2020). It is important to note that we did not always reach the target PLC levels (see Supplementary Table 1), but treatment implementation was successful in that we created a gradient in water availability with our treatments. Full details have been provided by Resco De Dios et al. (2020).

### Gas Exchange Measurements

Leaf gas exchange was measured with a portable photosynthesis system (LI-6400XT, Li-Cor Inc., Lincoln, NE, United States). We measured 3–5 plants in each treatment at two different periods during the night: between 23:00 h and 01:00 h and between 03:00 h and 05:00 h, and also during the day (10:00–13:00 h). We did not observe significant differences between the stomatal conductance measured over night at the different times (*p* = 0.79) and measurements were pooled together in subsequent analyses. To understand if measurement errors arising from low flux rates affected our measurements, we also conducted measurements with an empty chamber for 4–5 h, following previously published protocols (Resco De Dios et al., 2013). Results were always one or more orders of magnitude lower or negative. Given these results, we concluded that leaf observations were reliable and that a general correction was not required.

Block temperature was set at 25°C during the night and at 30°C during the day, CO_2_ at 400 ppm and relative humidity at ∼30%. This meant that *D* during nighttime measurements was at ∼2.2 kPa, which was substantially higher than that naturally occurring during the night (Resco De Dios et al., 2020). We chose this design to induce nocturnal stomatal closure and test whether *g*_*n*_ indicates *g*_*res*_.

During the daytime, we performed measurements at two different levels of PAR: 1,500 μmol m^–2^ s^–1^ and 40 μmol m^–2^ s^–1^. We first measured under growth PAR (1,500 μmol m^–2^ s^–1^ for plants in the sun treatment and 40 μmol m^–2^ s^–1^ for plants in the shadow treatment) and then at the other PAR level (40 μmol m^–2^ s^–1^ for plants in the sun treatment and 1,500 μmol m^–2^ s^–1^ for plants in the shadow treatment). The leaves were exposed for 10–20 min under the different light intensities until acclimation to the new light level. We only used data measured under growth PAR for analyses, and the rest was reserved for model validation. We note that a sudden exposure to 1,500 μmol m^–2^ s^–1^ for a plant growing in the shade would represent a sunfleck, and this could affect the performance of steady-state stomatal models (Way and Pearcy, 2012). When the leaf did not cover the chamber completely, it was scanned and we corrected measurements for leaf area.

In order to parameterize the photosynthesis component of the coupled leaf gas exchange model, we also measured the response of photosynthesis (*A*) to different internal CO_2_ concentrations (*C*_*i*_) following the protocols from Long and Bernacchi (2003). Briefly, we started measurements with an ambient CO_2_ concentration (*C*_*a*_) of 400 ppm and, after 5 min of acclimation, we sequentially changed *C*_*a*_ to 300, 250, 200, 150, 100, 50, 0, 400, 500, 650, 800, 1000, 1250, and 1500 ppm. These measurements were performed at saturating light (1,500 μmol m^–2^ s^–1^), setting block temperature at 30°C and with RH as high as we could achieve, which was ∼50%.

### Measurements of *g*_*MLD*_

*g*_*MLD*_ was measured as the mass loss of detached leaves following Phillips et al. (2010) in five leaves per treatment, weighting the leaves every 5 min during 2 h after collection. We wrapped the petiole with paraffilm so that only water lost through the leaf was measured. We performed the measurements in the laboratory, briefly after collection, where we monitored the temperature and relative humidity. Residual conductance was then calculated as:

(3) $$g_{\text{MLD}}=E_{\text{MLD}}PD^{-1}$$

where *E*_*MLD*_ is mass loss per projected leaf area (mol m^–2^ s^–1^), *P* is atmospheric pressure (kPa) and *D* is the vapor pressure deficit (kPa). *g*_0_ was defined as *g*_*MLD*_ when the leaf originated from the shade treatment at *P*_0_ (*P*_0__shade) and *g*_*min*_ was defined as *g*_*MLD*_ at *P*_80_ in the sun treatment (*P*_80__sun).

### Analyses of Non-structural Carbohydrates and Elemental Composition

To better understand the physiological mechanisms explaining variations in *g*_*res*_ with treatments, we analyzed the concentrations of non-structural carbohydrates, changes in leaf mass per area (LMA) and nitrogen concentrations (*N*_*mass*_). We collected all the leaves in five plants for each treatment. Immediately after collection, we scanned the leaves to measure the total area and they were then microwaved for 30 s and 700W to stop further metabolic processes. We then oven dried the samples (48 h in 105°C) and recorded the dry mass. Leaf area and dry weight was used to estimate LMA.

We followed previously developed protocols for extracting the percentage for sugar and starch (Palacio et al., 2007). This method consists of grinding the dried leaves with a mill (IKA A10, IKA-Werke, Staufen, Denmark) and making two extractions: one for extracting soluble sugars (sugars from now on) and a second extraction for starch. The first step of the sugar extraction consisted of adding 10 ml of ethanol (80% v/v) to 50 mg of sample, which we then left for 30 min at 60°C in water bath, and then we centrifuged (NEYA 8, REMI ELEKTROTECHNIK LTD., Vasai, India) the sample for 10 min at 3200 rpm. In the second step we added 50 μl of the supernatant, 450 μl of ethanol (80%), 500 μl of phenol (28%), and 2500 μl of sulfuric acid (96%), we shook the mix and let it stand for 30 min. In the third step we read the absorbance at 490 nm with spectrophotometer (Spectrophotometer UV-1600PC, VWR, Radnor, PA, United States) after removing the supernatant and drying the sample at 70°C during 16 h.

In the starch extraction, we added 4 ml of sodium acetate (pH 4.5) to the dry sample and left it for 60 min in a water bath (60°C). Once the sample cooled down, we added 1 ml of Amyloglucosidase (0.5% w/v) and we incubated the mix in the stove for 16 h at 50°C. We then added sample 50 μl of supernatant, 450 μl of sodium acetate (pH 4.5), 500 μl of phenol (28%), and 2,500 μl of sulfuric acid (96%). We then mixed it and let sit for 30 min, and then we measured the absorbance at 490 nm with the spectrophotometer.

We analyzed nitrogen concentration in an elemental analyzer (Carlo Erba 1110 Elemental Analyzer) at the University of Wyoming following previously published procedures (Hoffman et al., 2019).

### Statistical Analyses

We examined statistical differences across treatments in *g*_*MLD*_, *g*_*n*_, and *g*_*d*_ using an ANOVA (followed by Tukey’s HSD test) with species, light and water treatments as explanatory variables. Measurements of *g*_*MLD*_, were conducted on different individuals within a treatment. Consequently, we examined whether values were comparable within a given treatment by examining variation in the mean ± 95% CI in *g*_*MLD*_, *g*_*n*_, and *g*_*d*_.

To examine potential drivers of variation in *g*_*res*_, we additionally performed correlation analyses between conductance and NSC, LMA, gas exchange parameters and Ψ_*md*_.

All data was analyzed with R 3.6.3 (R Core Team, 2020) using base packages and, additionally, “corrplot” for plotting the correlation table (Wei and Simko, 2017).

### Modeling

In order to examine the effects of the different forms of measuring residual conductance over stomatal predictions and coupled photosynthetic responses, we performed two exercises. First, we examined the effects on stomatal predictions on different implementations of Eqs 1, 2. Second, we examined the effects of the different measured values of *g*_*res*_ on a photosynthesis-stomatal conductance coupled model.

For the first exercise, we compared the performance of different versions of the Ball-Berry (BB) model (Ball et al., 1987). First, we examined the version proposed by Duursma et al. (2019, BBD):

(4) $$g_{\text{s}}=\text{max}\left[\text{max}\left(g_{0},g_{\text{min}}\right),mA\mathrm{RH}/C_{\text{a}}\right]$$

and we used three different forms of the left hand term [max (*g*_0_, *g*_*min*_)]. That is, we compared model performance when the left hand term used *g*_0_ and *g*_*min*_ estimated from *g*_*MLD*_ (BBD_*MLD*_), *g*_*n*_ (BBD_*n*_), and *g*_*d*_ (BBD_*d*_). In all cases, *g*_0_ was defined as conductance (*g*_*MLD*_, *g*_*n*_, or *g*_*d*_, depending on the case) in the shade treatment without water stress (*P*_0__shade) and *g*_*min*_ as conductance in the sun under strong water stress (*P*_80__sun).

We compared these results with the original version of the Ball-Berry model (BB):

(5) $$g_{\text{s}}=g_{\text{int}}+mA\mathrm{RH}/C_{\text{a}}$$

where *g*_*int*_ and *m* were both estimated through least squares fitting.

Finally, we used an intermediate option where we used Eq. 5 but where *g*_*int*_ was replaced by actual *g*_*MLD*_ measurements (BB_meas_g_*MLD*_), instead of being estimated through least squares. We also tried with *g*_*n*_, in addition to *g*_*MLD*_, but differences were negligible, as will be discussed later in more detail.

Model calibration was performed with data collected under growth PAR (1,500 μmol m^–2^ s^–1^ for sun treatment and 40 μmol m^–2^ s^–1^ for the shade treatment). Model validation was performed with data collected under different PAR levels. That is, with PAR at 40 μmol m^–2^ s^–1^ for the sun treatment and at 1,500 μmol m^–2^ s^–1^ for the shade treatment. Model comparison was performed calculating the Akaike Information Criterion (AIC) and a model was considered more plausible when the AIC was smaller by a difference of 2 or more units (Burnham and Anderson, 2002). We also examined the variation in the slope, intercept and R^2^ of the observed *vs* predicted relationship.

For the second exercise, we simulated the effects of the different values of *g*_*MLD*_, *g*_*n*_ and *g*_*d*_ on predictions of *C*_*i*_ with varying PAR and on the effect temperature on leaf evaporation. We used the *A/C*_*i*_ curves to parameterize a coupled photosynthesis model (Duursma, 2015) and we conducted the simulation following previously published protocols (Duursma et al., 2019). We note that differences in mesophyll conductance across species and treatments could affect estimates of photosynthetic parameters (Flexas et al., 2012).

## Results

### Effects of Shade and Drought on *g*_*MLD*_ and *g*_*n*_

**FIGURE 1:**
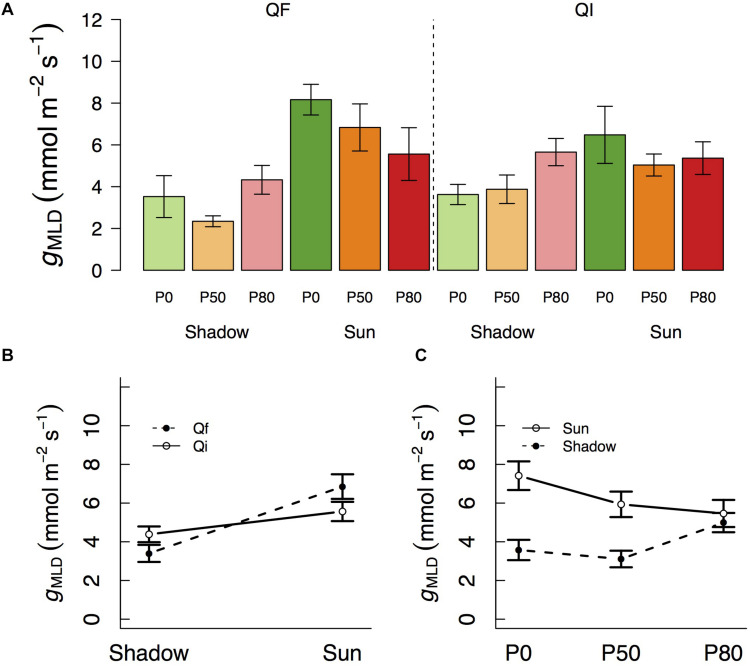
**(A)** Variation in residual conductance measured from the mass loss of detached leaves (g_*MLD*_) across different light treatments (shadow vs sun) and water treatments (P_0_, P_50_, and P_80_). Significant interactions across treatments are plotted in **(B,C)**. Bars indicate mean values per treatment and error bars indicate SE.

We observed that *g*_*MLD*_ varied significantly with species and light and also with light and water (Table 1 and Figure 1). The interactions between species and light resulted in *g*_*MLD*_ significantly declining from 6.9 in the sun to 3.4 mmol m^–2^ s^–1^ shadow in *Q. faginea*. However, *g*_*MLD*_ in *Q. ilex* did not differ across light levels (5.6 in the sun and 4.4 mmol m^–2^ s^–1^ in the shade). The interaction between light and water was such that *g*_*MLD*_ declined with drought in the sun treatment (from 7.4 at P_0_ to 5.5 mmol m^–2^ s^–1^ at P_80_), but *g*_*MLD*_ increased with drought in the shade from 3.1 at P_50_ to 5.0 mmol m^–2^ s^–1^ at P_80_).

**TABLE 1:** ANOVA Table on the effects of species, light treatment, water treatment on residual conductance measured from the mass loss of detached leaves (*g*_*MLD*_), from nocturnal conductance (*g*_*n*_), and also from daytime conductance (*g*_*d*_).

Factor	Df	*F*	*P*-value
***g*_*MLD*_**			
Species	1	0.12	0.73
Light	1	23.4	< 0.0001
Water	2	1.35	0.27
Species × Light	1	5.41	0.02
Species × Water	2	0.75	0.48
Light × Water	2	4.04	0.02
Species × Light × Water	2	0.35	0.71
***g*_*n*_**			
Species	1	0.89	0.35
Light	1	1.27	0.26
Water	2	2.14	0.12
Species × Light	1	4.98	0.03
Species × Water	2	2.53	0.09
Light × Water	2	0.14	0.87
Species × Light × Water	2	0.20	0.82
***g*_*d*_**			
Species	1	5.84	0.02
Light	1	51.21	< 0.001
Water	2	138.66	< 0.001
Species × Light	1	16.99	< 0.001
Species × Water	2	1.08	0.34
Light × Water	2	91.74	< 0.001
Species × Light × Water	2	21.24	< 0.001

**FIGURE 2:**
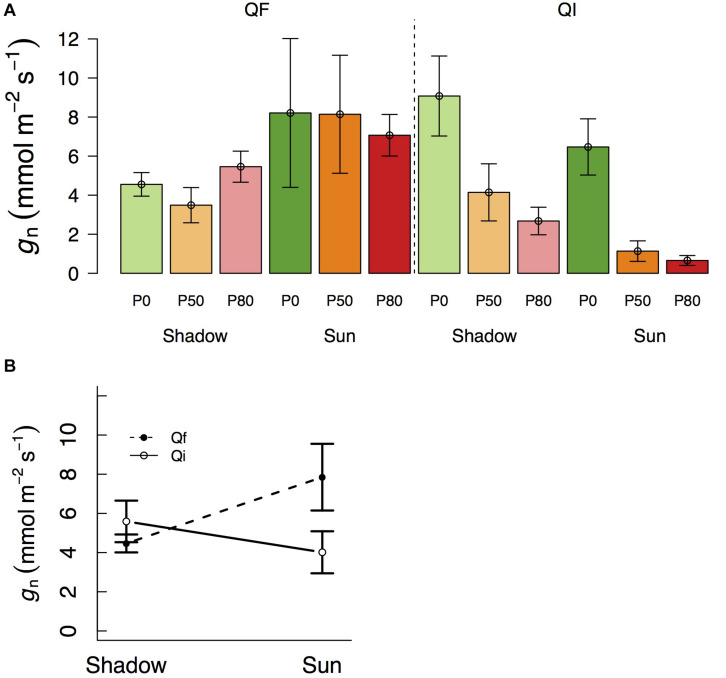
**(A)** Variation in residual conductance measured from nocturnal conductance under high *D* (g_*n*_) across different light treatments (shadow vs sun) and water treatments (P_0_, P_50_, and P_80_). Significant interactions across treatments are plotted in **(B)**. Bars indicate mean values per treatment and error bars indicate SE.

Variation in *g*_*n*_ followed a pattern of variation similar to that of *g*_*MLD*_ in that it also varied significantly with species and light treatments (Table 1 and Figure 2). *g*_*n*_ was not different between species at the shade treatment (4.5 and 5.6 mmol m^–2^ s^–1^ in *Q. faginea* and *Q. ilex*, respectively), but there was a significant increase in *g*_*n*_ in *Q. faginea* (7.8 mmol m^–2^ s^–1^) in the sun treatment. Instead, *g*_*n*_ in *Q. ilex* at the sun treatment was similar to that in the shade (4.0 mmol m^–2^ s^–1^; Figure 1B). Differences across water treatments were not significant.

### Effects of Shade and Drought on *g*_*d*_

**FIGURE 3:**
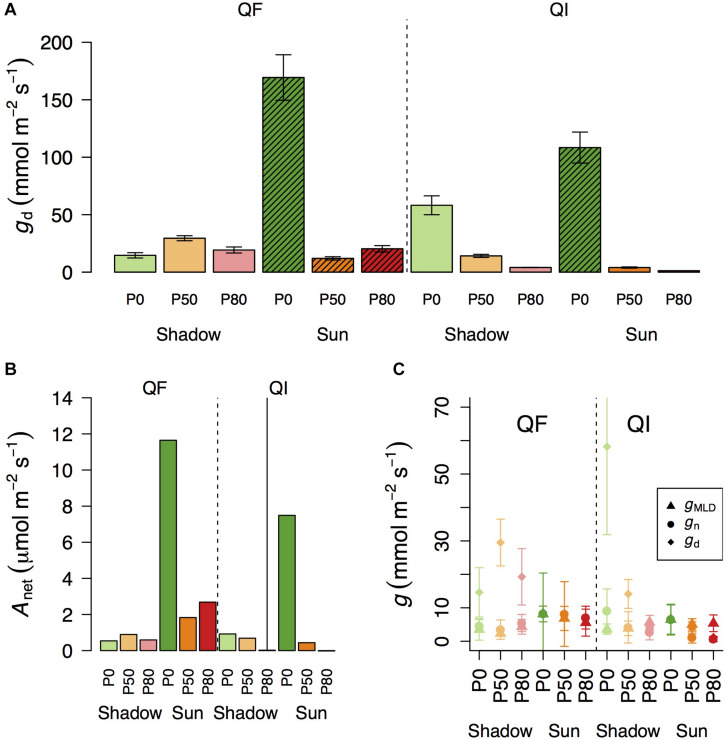
Variation in daytime leaf conductance (**A**, *g*_*d*_) and net assimilation (**B**, *A*_*net*_) across different light treatments (shadow vs sun) and water treatments (P_0_, P_50_, and P_80_). Bars indicate mean values per treatment and error bars indicate SE. Hatched bars in **(A)** indicate that *A*_*net*_ in that treatment is significantly higher than 1. **(C)** Differences in residual conductance as measured by from the mass loss of detached leaves (g_*MLD*_), from nocturnal conductance under high *D* (*g*_*n*_), or from *g*_*d*_ when *A*_*net*_ was smaller than 1 [note that some values of *g*_*d*_ are missing in **(C)** if *A*_*net*_ for that treatment was higher than 1]. Error bars indicate 95% CI.

Of particular relevance for this study is to examine *g*_*d*_ when *A*_*net*_ approaches zero (Figure 3B), so that one can test the potential use of *g*_*d*_ as an indicator of residual conductance. There are different definitions in the literature as to what is meant by photosynthesis approaching zero (Leuning, 1995; Barnard and Bauerle, 2013). Here we used g_*d*_ when *A*_*net*_ was at, or below, 1 μmol m^–2^ s^–1^. In *Q. faginea*, this occurred under the shade treatments at all water stress levels, where *g*_*d*_ varied between 14.6 and 29.5 mmol m^–2^ s^–1^ (Figure 3).

In *Q. ilex*, *A*_*net*_ was always below 1 μmol m^–2^ s^–1^ in the shadow treatments at all water stress levels. However, there was significant variation in *g*_*d*_ as it varied from 58 mmol m^–2^ s^–1^ in P_0_ to 14 and 4 mmol m^–2^ s^–1^ in P_50_ and P_80_, respectively. Within the sun treatments, *A*_*net*_ was always below 1 under water stress (at P_50_ and P_80_) where *g*_*d*_ varied between 4 and 1 mmol m^–2^ s^–1^, respectively. *g*_*d*_ under water stress (P_50_ and P_80_) was not different between shadow and sun treatments (Figures 3A,B).

### Differences Between *g*_*MLD*_, *g*_*n*_, and *g*_*d*_

Within a given treatment, *g*_*n*_ was indistinguishable from *g*_*MLD*_: 95% CI error bars always overlapped (Figure 3C). In *Q. faginea*, values of *g*_*MLD*_ were usually below those of *g*_*n*_, but the absolute difference was less than 4 mmol m^–2^ s^–1^. In *Q. ilex*, the difference between *g*_*n*_ and *g*_*MLD*_ was less than 1 mmol m^–2^ s^–1^.

In contrast, *g*_*d*_ was consistently and significantly above both, *g*_*MLD*_ and g_*n*_ in *Q. faginea*. It should be noted that, for this comparison, we only used *g*_*d*_ when *A*_*net*_ was below 1 μmol m^–2^ s^–1^. That is, we did not seek to compare values of *g*_*d*_ with *g*_*MLD*_ and *g*_*n*_ if *A*_*net*_ was above 1 μmol m^–2^ s^–1^ because, in that case, photosynthesis does not tend to zero. The average difference of *g*_*d*_ with *g*_*MLD*_ was 17.7 mmol m^–2^ s^–1^ and the average difference of *g*_*d*_ with *g*_*n*_ was 10 mmol m^–2^ s^–1^. The only case in which *g*_*d*_ was not different from *g*_*MLD*_ and *g*_*n*_ was in the sun treatments in *Q. ilex*.

### Correlates Explaining Variation in *g*_*MLD*_

**FIGURE 4:**
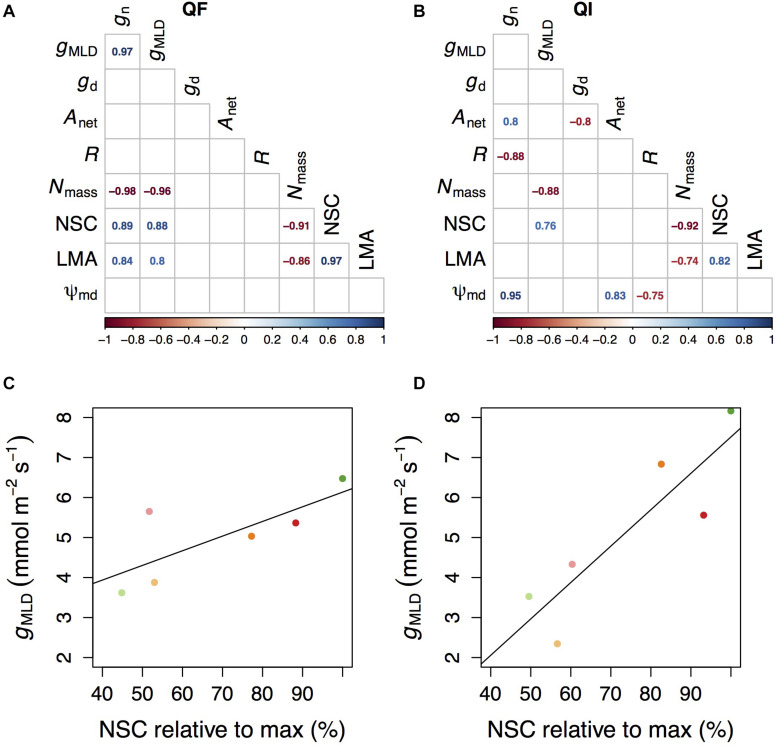
Correlation table between the measured parameters in *Q. faginea* (Qf, **A**) and *Q. ilex* (Qi, **B**). Relationship between conductance measured as mass loss of detached leaves under and non-structural concentrations (NSC) in Qf **(C)** and Qi **(D)**. NSC are provided relative to the maximum values measured in our experiment. Values in **(C,D)** indicate mean per treatment, and each treatment is indicated by a different color following the same convention as in Figures 1–3.

Overall, the relationships between the different indicators of *g*_*MLD*_ and other physiological parameters were species-specific (Figure 4A). The only exceptions were *N*_*mass*_ and NSC concentrations which had a negative and a positive correlation, respectively, with *g*_*MLD*_ in both species (Figure 4). In turn, *N*_*mass*_ correlated negatively with NSC concentrations and LMA in both species. NSC also correlated with LMA in both species, albeit positively. In *Q. faginea*, *g*_*MLD*_ and *g*_*n*_ also correlated positively with LMA and *g*_*MLD*_ also correlated positively. In *Q. ilex*, *g*_*n*_ showed a negative correlation with respiration (*R*) and a positive correlation with Ψ_*md*_ and with *A*_*net.*_ NSC concentrations were negatively affected by the shade treatment (Supplementary Figure 1A).

### Modeling *g*_*d*_: Comparing Different Formulations of the BB Model

We first compared the performance of the model proposed by Duursma et al. (2019) when *g*_0_ and *g*_*min*_ had been defined on the basis of *g*_*MLD*_ (BBD_*MLD*_), of *g*_*n*_ (BBD_*n*_), and of g_*d*_ (BBD_*d*_). In all cases, the original *g*_0_ and *g*_*min*_ were defined as the level conductance (*g*_*MLD*_, *g*_*n*_, or *g*_*d*_, depending on case) in the P_0__shade treatment (low light) and in the P_80__sun treatment, respectively, (high water stress).

Model performance was superior when the model was based on *g*_*MLD*_ (BBD_*MLD*_), but differences with the model based on *g*_*n*_ were minor (ΔAIC = 0.3 for *Q. faginea* and 2.3 for *Q. ilex*). However, the model based on *g*_*d*_ showed consistently a larger AIC, indicating smaller plausibility (Table 2).

**TABLE 2:** Model comparison. We compared for *Q. faginea* (QF) and *Q. ilex* (QI) different models based on the Akaike Information Criterion (AIC), the change in AIC relative to the lowest (ΔAIC) and the *R*^2^, slope and intercept of the observed vs predicted relationship. For the slope and intercept we show the mean value (and SE).

	*g*_*int*_ (mmol m^–2^ s^–1^)	AIC	Δ AIC	*R*^2^	Slope		Intercept	
***QF***								
BBD_*MLD*_	5.5	–106.88	0	0.88	1.04	(0.09)	0.001	(0.005)
BBD_*n*_	7.1	–106.6	0.28	0.87	1.06	(0.09)	0.0006	(0.005)
BBD_*d*_	20.3	–102.32	4.56	0.84	*1.23*	*(0.13)*	−*0.013*	*(0.007)*
BB^1^	8.9	–102.3	4.58	0.85	1.02	(0.09)	–0.01	(0.006)
BB_meas_*g*_*MLD*_	5.5	–104.3	2.58	0.86	0.99	(0.09)	0.002	(0.005)
***QI***								
BBD_*MLD*_	5.3	–98.52	1.64	0.97	**1.2**	**(0.05)**	–0.0001	(0.003)
BBD_*n*_	9.1	–96.22	3.94	0.97	**1.2**	**(0.05)**	–0.002	(0.008)
BBD_*d*_	58.2	–69.92	30.24	0.85	**1.6**	**(0.17)**	**−0.067**	**(0.01)**
BB^1^	16.8	–98.14	2.02	0.98	**1.3**	**(0.05)**	**−0.018**	**(0.004)**
BB_meas_*g*_*MLD*_	5.3	–100.16	0	0.98	**1.2**	**(0.04)**	–0.001	(0.003)

*The models compared include the Ball-Berry model (Eq. 5, BB), the BB model where the intercept is measured, rather than estimated (BB_meas_g_MLD_) and the modification of the Ball-Berry model proposed by Duursma (Eq. 4, BBD) using g_MLD_ (BBD_MLD_), g_n_ (BBD_n_), or g_d_ (BBD_d_) as g_0_ and g_min_. We also provide the values of the intercept, g_int_ (the maximum between g_0_ and g_min_ in the first three models), that were used in each case. Values in bold (or italics) in slope and intercept indicate values significantly (or marginally) different from 1 and 0, respectively. ^1^Note that g_int_ in this case is estimated through least squares, rather than measured as in the other models.*

We compared the performance of these three models against the original Ball- Berry (BB) and we observed that BBD_*MLD*_ and BBD_*n*_ performed better only in *Q. faginea*, where the difference in AIC was bigger than 4. For *Q. ilex*, however, the AIC was similar across models although the intercept of the observed *vs* predicted relationship was significantly different from 0 only in the BB model.

Finally, we compared the performance of the Ball-Berry model but where, instead of fitting *g*_*int*_ through least squares, we use actual *g*_*MLD*_ measurements (BB_meas_*g*_*MLD*_), which we defined originally as *g*_*MLD*_ under water stress (P_80__sun). We observed that this was the best model in *Q. ilex* as it had the smallest AIC although the difference was not significant with BBD_*MLD*_ (ΔAIC = 1.64). In *Q. faginea*, BB_meas_*g*_*MLD*_ peformed worst than BBD_*MLD*_ (ΔAIC = 2.6).

Differences between BB_meas_*g*_*MLD*_ were significant with the BB model (AIC = 2 for both species) and it was also more plausible than the BBD_*d*_ model in *Q. faginea* (AIC > 2). Differences between BB_meas_g_*int*_ and the other models were not significant. We tried fitting BB_meas_*g*_*int*_ with different values of *g*_*int*_ (e.g., using values under shade, or from *g*_*n*_), but differences were not significant (*data not shown*).

### Modeling *g*_*d*_: Coupled Leaf Gas Exchange Model

**FIGURE 5:**
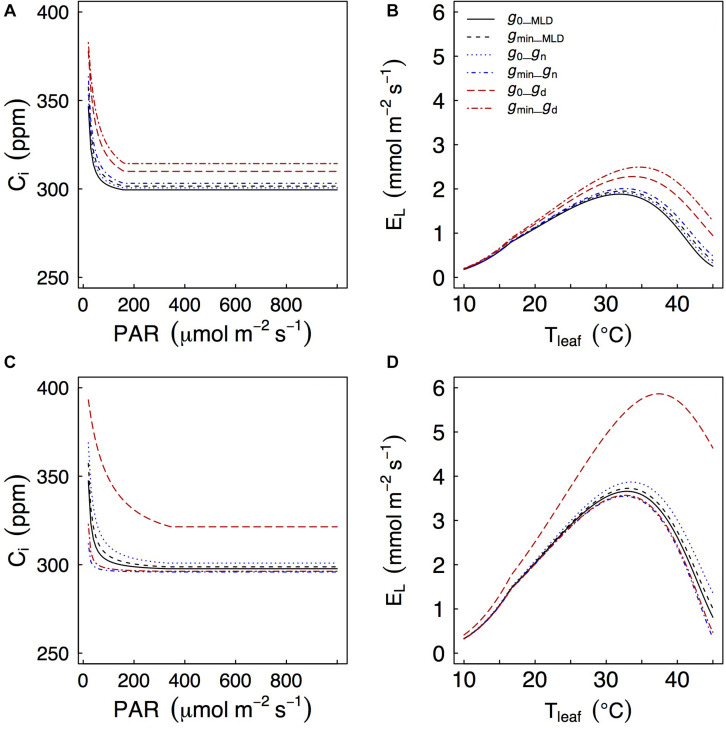
Effects of different values of *g*_*res*_ on a coupled photosynthesis model as in Duursma et al. (2019). Models are for *Q. faginea* **(A,B)** and *Q. ilex* **(C,D)**. *g*_0_ and *g*_*min*_ represent *g*_*res*_ under low light (P_0__shadow treatment) and water stress (P_80__sun treatment), respectively. Subscripts _*MLD*_, _*n*_, and _*d*_ indicate that *g*_0_ or *g*_*min*_ (depending on case) were estimated from mass loss of detached leaves, from nocturnal conductance, or from daytime conductance.

Depending on how *g*_*res*_ was measured, we found significant differences of simulated gas exchange. In particular, when *g*_*d*_ was used we always observed higher values of *C*_*i*_ at any PAR level and also higher leaf transpiration rates (*E*_*l*_) as temperatures increased because *g*_*d*_ was often larger than *g*_*MLD*_ and *g*_*n*_ (Figure 5).

Generally speaking, there was little difference in simulated *C*_*i*_ and *E*_*l*_ regardless of whether *g*_*MLD*_ or *g*_*n*_ were used, and whether they were defined from *g*_0_ or from *g*_*min*_. The only exception was that, in *Q. ilex*, there were some differences in predicted *C*_*i*_ (particularly at low PAR levels) and in predicted leaf transpiration (particularly at peak *E*_*l*_) when *g*_*res*_ was defined from *g*_*n*_: using *g*_*n*_ from the P_0__shade (*g*_0_) treatment led to higher predicted *C*_*i*_ and *E*_*l*_ than using *g*_*n*_ from the P_80__sun treatment (*g*_*min*_). It should be noticed that *g*_*n*_ from the P_0__shade treatment was one order of magnitude larger than *g*_*n*_ from the P_80__sun treatment (9.1 vs 0.6 mmol m^–2^ s^–1^, respectively).

## Discussion

We observed that residual conductance varied significantly across light and water treatments in an interactive (non-additive) fashion and the responses differed across species. There were no significant differences as to whether residual conductance was measured from *g*_*MLD*_ or from *g*_*n*_, but the values were significantly higher when using *g*_*d*_. *g*_*MLD*_ was positively correlated with NSC concentrations, suggesting that further reductions in *g*_*MLD*_ under drought may be limited by low NSC availability. From a modeling perspective, the small measured differences between *g*_*MLD*_ and *g*_*n*_ generally did not impact model performance. Although residual conductance differed significantly under experimental treatment, such differences in residual conductance showed only a moderate impact on model performance. That is, model performance did not critically depend upon whether residual conductance was measured under strong shade or under strong water stress. There was also little difference in model fit when either *g*_*MLD*_ or g_*n*_ were used as an absolute minimum in Eq. 4 (BBD_*MLD*_ or BBD_*n*_), or when they were used as the intercept of the BB equation (BB_meas_g_*MLD*_).

### Shade and Drought Interact as Drivers of *g*_*MLD*_ Although Responses Are Species-Specific

We observed that *g*_*MLD*_ declined under increasing drought in the sun treatment. In the shade treatment, however, *g*_*MLD*_ remained low and constant, regardless of the water treatment. This result indicates that drought only affects *g*_*MLD*_ under high light because, under shade, light limitations lower *g*_*MLD*_ to a minimum that is not affected by water stress. It is worth noting that, at least for some species, full acclimation after changes in the light growth environment may require more than one growing season (Aranda et al., 2001). In other words, the strong limitation imposed by the low light over *g*_*MLD*_ may increase even more in subsequent years.

Previous studies had identified how *g*_*MLD*_ often decreases under exposure to water stress and light, as a result of changes in wax composition, when each effect is examined in isolation (Shepherd and Wynne Griffiths, 2006). However, our experiment may be the first to examine *g*_*MLD*_ responses in a multifactorial experiment. Interestingly, light and water effects were not additive. That is, we did not observe a lower *g*_*MLD*_ under low light and high water stress, as would be expected from an additive effect of both factors.

The response to shade was, however, species-specific. *g*_*MLD*_ increased in the sun treatment only in the deciduous *Q. faginea*, whereas the increase in *Q. ilex g*_*MLD*_ under sun was not significant.

### *g*_*MLD*_ Correlated With Low NSC Concentrations

We can speculate that the reason why *g*_*MLD*_ was not lower under the high water stress and shade treatment (relative to other shade treatments under less water stress) is related to carbohydrate limitations. We observed a significant and positive correlation between NSC and *g*_*MLD*_ across species. A synthesis of variation in NSC across species reports that a minimum NSC of 46% is always conserved (Martínez-Vilalta et al., 2016). In our results we also observed a minimum NSC that was close to the 46% of the maximum NSC that we measured (Figures 4C,D).

A possible explanation on why *g*_*MLD*_ did not decrease further under the joint drought and shade stress is related to a lack of NSC to feed the building of additional wax and/or epidermal layers. That is, once plants have reached the minimum NSC threshold of 46% relative to maximum, they will seek to preserve their NSC for other functions, such as osmoregulation, at the expense of building thicker cuticles or additional wax layers. We note that osmoregulation under shade may be impaired in oaks (Aranda et al., 2005; Rodriguez-Calcerrada et al., 2010).

At any rate, this is the first study, to our knowledge to raise this possibility. This result should thus be interpreted with caution. We acknowledge that the correlation between *g*_*MLD*_ and NSC may have been affected by jointly considering plants under different light and water regimes. Subsequent work would thus be needed to confirm this hypothesis.

### Residual Conductance in Relation to Respiration, LMA and *N*_mass_

Despite stomatal closure, *g*_*res*_ did not limit CO_2_ diffusion out of the leaf. In fact, there was a negative correlation between nocturnal conductance and respiration in one of our (*Q. ilex*) species, indicating higher CO_2_ efflux at lower *g*_*n*_ and, consequently, that reduced *g*_*n*_ levels were far from limiting respiration. This contradicts earlier studies that cytotoxic CO_2_ build-up could occur under nocturnal stomatal closure (Fricke, 2019) but it aligns along with the results of modeling, indicating that only under conductances that are orders of magnitude lower to those reported here could a cytotoxic CO_2_ build-up occur (Resco De Dios et al., 2019).

*g*_*MLD*_ increased in the sun in *Q. faginea.* LMA also increased with light (*data not shown*) and it was significantly correlated with *g*_*n*_ and with *g*_*MLD*_ in *Q. faginea*. LMA is an indicator of the degree of sclerophylly, which could serve to decrease residual conductance by increasing cuticle thickness. However, LMA also increased with light in the sclerophyll *Q. ilex*, where LMA did not correlate with *g*_*n*_ or *g*_*MLD*_. This result matches with previous studies in *Quercus* indicating that any effects of LMA in *g*_*n*_ and *g*_*MLD*_ may be modified by changes in the cuticle composition (Bueno et al., 2020). We note that this argument is speculative and based only on circumstantial evidence.

*N*_*mass*_ showed a negative correlation with *g*_*MLD*_ in both species, and with *g*_*n*_ in *Q. faginea*. This result indicates that species with a higher photosynthetic investment will decrease the investment in residual conductance. This points toward a potential mechanism underlying the trade-off between investment for C uptake (higher *N*_*mass*_) and preventing catastrophic water losses (reduced *g*_*MLD*_). Further studies will be necessary to test the generality of this hypothesis.

### Residual Conductance May Be Measured via *g*_*MLD*_ or via *g*_*n*_ Under High *D*

Measurements of nocturnal conductance under the relatively high *D* from this experiment were statistically indistinguishable from independent measurements of residual conductance indicating that the latter was driving the former. It had been previously argued that measurements of *g*_*n*_ would not be valid indicators of residual conductance, because *g*_*n*_ is actively regulated (Duursma et al., 2019). Our results suggest that this argument from Duursma et al. (2019) may only be valid when *g*_*n*_ is measured under low *D*.

Previous studies document that stomata often reach complete closure (or as complete as it can be) under lower *D* in the night, than in the day (Barbour and Buckley, 2007). This phenomenon would explain why *g*_*n*_ was much lower than *g*_*d*_ although *D* was comparable, and it is likely explained by the capacity of stomata to sense and open in response to light.

We also show how modeling results were not affected by either using *g*_*MLD*_ or *g*_*n*_. This result, however, needs to be interpreted with caution. We only focused on BB-type stomatal models and other results may be obtained in different model types. For instance, as Duursma et al. (2019) noted, changing minimum conductance from 2 to 4 mmol m^–2^ s^–1^ halved the time to reach mortality in a hydraulics model because it doubled the water losses (Duursma et al., 2019). Although differences were statistically not significant between *g*_*MLD*_ and *g*_*n*_, we still observed differences in mean residual conductance of 4 mmol m^–2^ s^–1^ across measurements, indicating that measurement errors and other sources of uncertainty may play a large for other model types, such as mortality models.

### Modeling *g*_*s*_

We acknowledge our dataset was limited to thoroughly test the best form of the BB model: we sampled under highly contrasting light and water conditions, but only once in time. We would thus need data over more time periods for a more thorough evaluation. However, our dataset allows for the development of some hypotheses, which may be expanded in subsequent studies.

We observed that there were only little differences between Eq. 5, where the original BB function was used but including measured *g*_*MLD*_ (BB_meas_g_*MLD*_), instead of the version proposed by Duursma et al. (2019; Eq. 4). Duursma et al. (2019) note that residual conductance acts as an actual minimum in the function they propose. However, if the goal is to use residual conductance as an actual stomatal minimum, one could consider the following equation instead:

(6) $$g_{\text{s}}=\text{max}\left(\text{min}\left({\text{g}}_{0},{\text{g}}_{\text{min}}\right),mA\mathrm{RH}/\text{Ca}\right)$$

where the minimum between *g*_0_ and *g*_*min*_ is chosen [not the maximum, as proposed by Duursma et al. (2019)].

At any rate, we did not observe major differences in model performance between the BBD model or Eq. 5. This result indicates that it is unlikely that losses in model performance will derive from the adoption of the alternative model formulations, as proposed by previous studies (De Kauwe et al., 2015; Duursma et al., 2019).

Our results also indicate that *g*_*MLD*_ and *g*_*n*_ can both be interchangeably, and that the choice between *g*_0_ and *g*_*min*_ exerts negligible consequences for model fitting. Earlier studies indicate a major effect of *g*_*res*_ (De Kauwe et al., 2015; Duursma et al., 2019). This is because those studies used a wide range of *g*_*res*_ values (10–40 mmol m^–2^ s^–1^, depending on the case), much higher that the variability we reported here when using *g*_*MLD*_ and *g*_*n*_ across treatments (Table 2). Synthesis studies similarly indicate limited variation in *g*_*res*_ within a family (Duursma et al., 2019). After discarding *g*_*d*_ as a reliable indicator of *g*_*res*_, our results indicate a minor effect of different methods and approaches used for measuring *g*_*res*_ and for modeling water use, at least in our two closely related species.

## Data Availability Statement

The raw data supporting the conclusions of this article will be made available by the authors, without undue reservation.

## Author Contributions

VR designed the experiment. CA performed research with the help of FC. VR analyzed the data and wrote the manuscript with important feedback from all co-authors. All authors contributed to the article and approved the submitted version.

## Conflict of Interest

The authors declare that the research was conducted in the absence of any commercial or financial relationships that could be construed as a potential conflict of interest.

## Supplementary Material

Supplementary Figure 1A Variation in leaf non-structural carbohydrate concentrations measured across different light treatments (shadow vs sun) and water treatments (P_0_, P_50_, and P_80_) in *Quercus faginea* (QF) and *Q. ilex* (QI). Bars indicate mean values per treatment and error bars indicate SE.

Supplementary Table 1A Target midday water potential (Ψ_*md*_) to reach the desired PLC according to Esteso-Martínez et al. (2006) for Q. *faginea* and to Peguero-Pina et al. (2014) for *Q. ilex*, and actual values. Mean (and SE) actual values are presented. The letters in “Actual PLC” indicate the results of *post hoc* analyses (Tukey HSD). This is a reproduction (with permission from the publisher) of Table 1 originally published in Resco De Dios et al. (2020).
