# Supplementary material for: Radiation and Drought Impact Residual Leaf Conductance in Two Oak Species With Implications for Water Use Models
Source: Front Plant Sci. 2020 Nov 27;11:603581. doi: 10.3389/fpls.2020.603581 (PMC7732681; doi:10.3389/fpls.2020.603581)
Supplement: Supplementary Figure 1A — Variation in leaf non-structural carbohydrate concentrations measured across different light treatments (shadow vs sun) and water treatments (P0, P50, and P80) in Quercus faginea (QF) and Q. ilex (QI). Bars indicate mean values per treatment and error bars indicate SE. [file Data_Sheet_1.docx]

**Supplementary Figure 1A |** Variation in leaf non-structural carbohydrate concentrations measured across different light treatments (shadow vs sun) and water treatments (P_0_, P_50_ and P_80_) in *Quercus faginea* (QF) and *Q. ilex* (QI). Bars indicate mean values per treatment and error bars indicate SE.
