# Supplementary material for: Radiation and Drought Impact Residual Leaf Conductance in Two Oak Species With Implications for Water Use Models
Source: Front Plant Sci. 2020 Nov 27;11:603581. doi: 10.3389/fpls.2020.603581 (PMC7732681; doi:10.3389/fpls.2020.603581)
Supplement: Supplementary Table 1A — Target midday water potential (Ψmd) to reach the desired PLC according to Esteso-Martínez et al. (2006) for Q. faginea and to Peguero-Pina et al. (2014) for Q. ilex, and actual values. Mean (and SE) actual values are presented. The letters in “Actual PLC” indicate the results of post hoc analyses (Tukey HSD). This is a reproduction (with permission from the publisher) of Table 1 originally published in Resco De Dios et al. (2020). [file Table_1.DOCX]

# Supplementary Table 1A | Target midday water potential (Ψ_md_) to reach the desired PLC according to Esteso-Martínez *et al.* (2006) for Q*. faginea* and to Peguero-Pina *et al.* (2014) for *Q. ilex*, and actual values. Mean (and SE) actual values are presented. The letters in “Actual PLC” indicate the results of post-hoc analyses (Tukey HSD). This is a reproduction (with permission from the publisher) of Table 1 originally published in Resco de Dios et al. (2020).

| Species | Light treatment | Water treatment | Target Ψ_md_ (MPa) | Target PLC (%) | Actual Ψ_md_ (MPa) | Actual PLC (%) |
| --- | --- | --- | --- | --- | --- | --- |
| *Q. ilex* | Sun | P_0_ | > -4 | < 20 | -1.3 (0.20) | 11.9 (2.3)**^c^** |
|  |  | P_50_ | -6 | 50 | -5.4 (0.54) | 64.7 (6.87)**^a^** |
|  |  | P_80_ | -8.9 | 80 | -6.4 (0.62) | 69.1 (7.56)**^ab^** |
|  | Shade | P_0_ | > -4 | < 20 | -0.8 (0.14) | 12.3 (2.84) **^c^** |
|  |  | P_50_ | -6 | 50 | -2.9 (0.77) | 41.2 (10.9)^abc^ |
|  |  | P_80_ | -8.9 | 80 | -4.4 (0.56) | 54.3 (10.69)^ab^ |
| *Q. faginea* | Sun | P_0_ | > -2.8 | < 20 | -1.1 (0.22) | 15.6 (2.73)**^c^** |
|  |  | P_50_ | -3.9 | 50 | -3.9 (0.28) | 53.2 (8.04)^ab^ |
|  |  | P_80_ | -5.5 | 80 | -4.2 (0.30) | 66.1 (9.89)**^a^** |
|  | Shade | P_0_ | > -2.8 | < 20 | -0.7 (0.12) | 7.5 (1.35) **^c^** |
|  |  | P_50_ | -3.9 | 50 | -1.1 (0.21) | 27.9 (9.86)**^bc^** |
|  |  | P_80_ | -5.5 | 80 | -2.6 (0.51) | 39.8 (13.0)**^abc^** |

|  |  |  |
| --- | --- | --- |
